# Time‐Resolved SAXS Reveals Distinct Millisecond Metal‐Induced Conformational Dynamics of Monomeric α‐Synuclein

**DOI:** 10.1002/advs.202512293

**Published:** 2026-02-27

**Authors:** Rebecca Sternke‐Hoffmann, Miriam Dos Santos Pinto, Xue Wang, Jinghui Luo

**Affiliations:** ^1^ PSI Center for Life Sciences Villigen PSI Switzerland

**Keywords:** α‐synuclein, conformational dynamics, intrinsically disordered proteins, metal ions, protein‐metal interaction, time‐resolved SAXS

## Abstract

Transition metal ions have been implicated in modulation the conformational behavior and aggregation of WT α‐synuclein (WT‐αSyn), associated with Parkinson's disease pathology. Nevertheless, the initial structural rearrangements that drive aggregation are not fully understood. Here, we employed time‐resolved small‐angle X‐ray scattering (TR‐SAXS) in a microfluidic setup to investigate the structural dynamics of monomeric WT‐αSyn upon interaction with Mn^2^^+^, Fe^3^^+^, Cu^2^^+^, and Zn^2^^+^. Using Guinier analysis, GNOM, and Ensemble Optimization Method (EOM), we resolved distinct, metal‐specific conformational transitions on the sub‐second timescale. Fe^3^^+^ induced rapid and sustained compaction of αSyn, while Cu^2^^+^ promoted extended and heterogeneous conformations, expanding the C‐terminal domain, and disrupting global folding. In contrast, Mn^2^^+^ and Zn^2^^+^ led to more gradual, domain‐specific compaction. Fractal dimension analysis and hierarchical clustering further revealed Fe^3^^+^ and Zn^2^^+^ enriched compaction states, while Cu^2^^+^ favored intermediate species potentially linked to early aggregation. These findings highlight how metal ion binding differentially and initially reshape the conformation ensemble of WT‐αSyn, offering mechanistic insight into metal‐induced misfolding pathways relevant to synucleinopathies.

## Graphical abstract

Time‐resolved SAXS reveals how physiologically relevant metal ions shape the earliest conformational responses of monomeric α‐synuclein. Fe^3^^+^ induces rapid compaction, Cu^2^^+^ generates heterogeneous and partially folded ensembles, while Mn^2^^+^ and Zn^2^^+^ cause only modest changes. The real‐time structural fingerprints provide new insight into how metal dyshomeostasis may steer α‐synuclein toward pathogenic aggregation pathways.

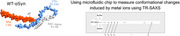

## 1 Introduction

Small‐angle X‐ray scattering (SAXS) is a powerful tool for examining the overall shape, size, and oligomerization state of biological macromolecules under near‐native conditions [1, 2]. Unlike other structural methods, SAXS does not require crystallization or labeling [3, 4], making it particularly useful for investigating dynamic and flexible systems such as intrinsically disordered proteins (IDPs). Given that approximately 40% of proteins in the human genome contain at least one disordered region and 25% are predicted to be completely disordered [5], SAXS has become crucial for characterizing their structural ensembles.

IDPs, including α‐synuclein (αSyn), lack a stable folded structure and exist as dynamic conformational ensembles in solution [6, 7, 8]. SAXS measurements provide insight into the conformational properties of such proteins by capturing their time‐averaged scattering profile that represents contributions from all accessible structural states [9, 10, 11]. For IDPs, the absence of a well‐defined low‐angle peak and the characteristic plateau in the Kratky plot reflect their extended and heterogeneous nature [10, 12]. Additionally, SAXS enables the analysis of conformational polydispersity, which is essential for understanding proteins with flexible linkers, unfolded states, or transition states between functional and aggregated forms [13, 14].

The ensemble optimization method (EOM) is particularly suited for SAXS‐based IDP studies [1, 10, 12]. EOM involves generating a large, random pool of potential conformations covering the available conformational space, computing their scattering profiles, and selecting an optimized subset that best fits the experimental data [2]. This approach has been instrumental in resolving the conformational ensembles of IDPs like αSyn, which exhibit a balance between compact and extended states. SAXS studies have shown that the radius of gyration (R_g_) of native αSyn (∼40 Å) is larger than that of a typical globular protein of similar size but smaller than that of a fully unfolded random coil (∼52 Å) [15]. This suggests that αSyn exists as a distribution of partially collapsed conformations rather than a completely random coil [11].

**FIGURE 1:**
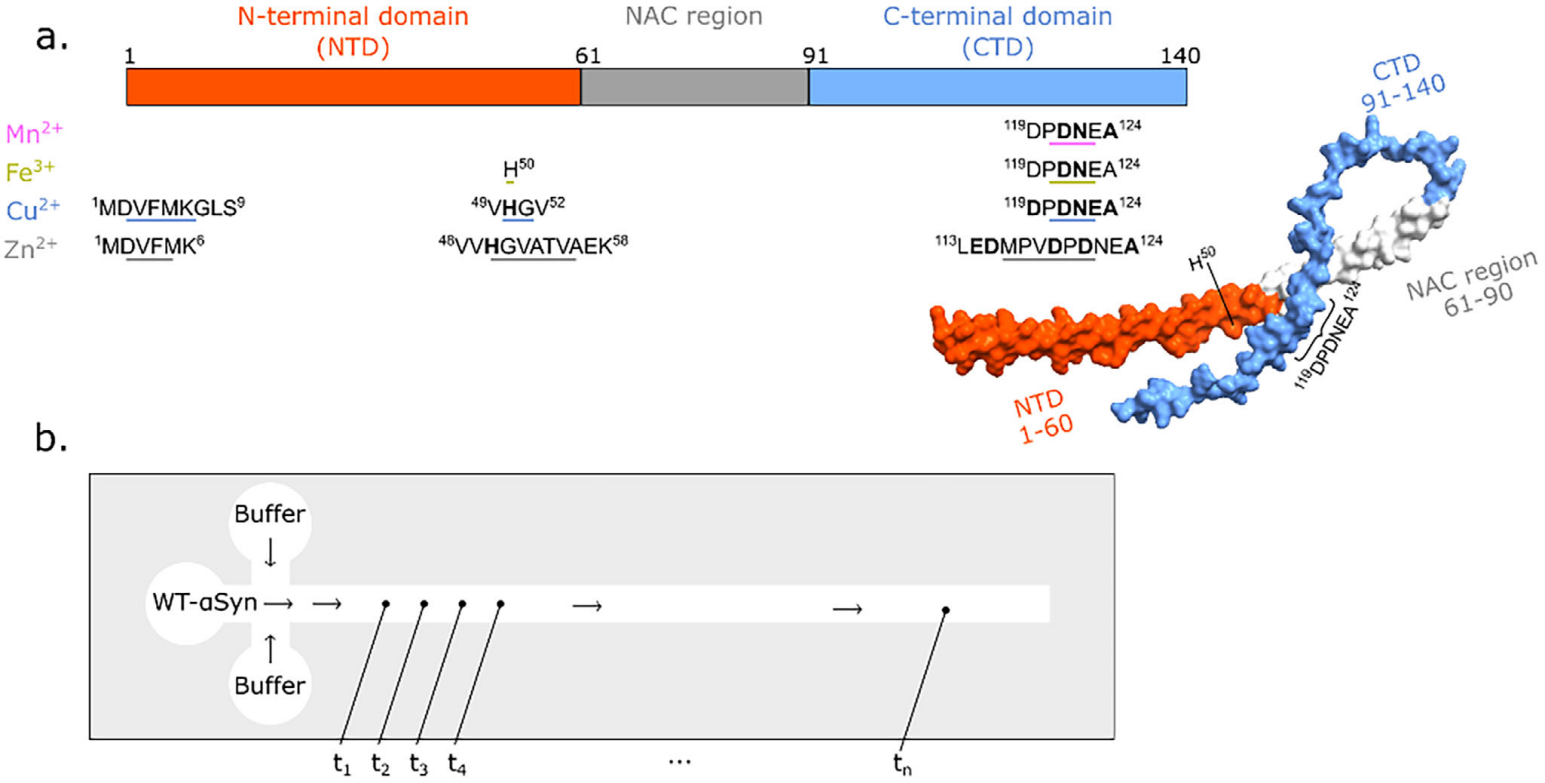
Experimental set‐up. (a) Schematic structure of WT‐αSyn with the three domains highlighted by color. The binding sites of different transition metal ions are marked, and the proposed main anchor residues are highlighted in bold. Structure was created with AlphaFold and visualized with ChimeraX. Alphafold predicts the NTD with an α‐helical folding since the helix induced by lipids is the only high‐resolution structure of monomeric WT‐αSyn available [45, 46]. (b) Exemplary drawing of the microfluidic chip. WT‐αSyn is pumped through the middle channel and mixed with the buffer (with and without metal ions) from both sides. SAXS spectra are recorded at defined points along the microfluidic channel.

Understanding the initial dynamics of αSyn and how pathological factors influence the structural equilibrium is essential for elucidating its role in neurodegenerative diseases pathogenesis, such as Parkinson's disease (PD). αSyn plays a key role in synaptic function, but the aggregation of αSyn into cytotoxic oligomers and amyloid fibrils leads to neurodegeneration [16, 17, 18, 19]. αSyn consists of three distinct domains: the highly conserved N‐terminal domain (NTD), the hydrophobic non‐amyloid component (NAC) region, and the flexible C‐terminal domain (CTD) [20] (see Figure 1a for an illustration). Particularly, the long‐range interactions between NTD and CTD demonstrate the potential to modulate αSyn aggregation [20, 21, 22, 23] due to protection of the NAC region, which builds the fibril core [24]. A key factor influencing the structural equilibrium of αSyn is the presence of transition metal ions, which have been implicated in modulating protein aggregation. Transition metal ions such as Mn^2^^+^, Fe^3^^+^, Cu^2^^+^, and Zn^2^^+^ are strongly implicated in Parkinson's disease pathology [25, 26]. Among them, Fe^3^^+^ and Cu^2^^+^ are the most extensively studied, as their redox‐active nature can drive oxidative stress and accelerate αSyn aggregation [27, 28, 29, 30]. In contrast, Zn^2^^+^, although essential and typically protective [31], exhibits disrupted homeostasis in the PD brain and binds αSyn with high affinity [32], providing an informative non–redox‐active comparison. Moreover, these metal ions display distinct binding preferences across different regions of the αSyn protein, underscoring their diverse mechanistic roles. For example, Cu^2^^+^ displays high‐affinity binding at substoichiometric levels at the NTD residues 1–9 and residues 49–52, where His50 is the main anchor, but more than two copper ions per monomer can be ligated via nonspecific electrostatic interaction with charged amino side groups, mainly involving the residues Asp119, Asp121, Asn122 and Glu123 in the CTD [33, 34, 35]. Zn^2+^ binding sites are also located in the NTD and CTD, mainly involving aspartate and glutamate [32, 36], while Mn^2+^ and Fe^3+^ bind primarily to the CTD involving residues 119–124. His50 can bind Fe^3+^ as well, particularly at higher Fe^3+^ concentrations (molar ratio 1:5) and in a fibril conformation [28, 37, 38, 39, 40, 41]. Metal binding may balance the negative charges in the CTD (net charge of −12) by electrostatic shielding, affecting its conformational dynamics and oligomerization propensity. The structure of αSyn including the metal ion binding sites of Mn^2+^, Fe^3+^, Cu^2^^+^, and Zn^2+^ is depicted in Figure 1a. All investigated metal ions are known to accelerate αSyn fibrillation in vitro. Elevated concentrations of these metals have been observed in PD pathology, where they may contribute to oxidative stress and protein misfolding [42, 43]. SAXS studies combined with molecular dynamics simulations have demonstrated that Cu^2^^+^ binding promotes a shift toward compact conformations, a structural transition associated with the formation of toxic oligomeric species [44], whereas previous SAXS measurements did not reveal a significant collapse to more compact structures based on R_g_ and Kratky analysis [28]. However, because these metal ions can rapidly trigger aggregation, conventional static SAXS and other biophysical techniques often capture only the aggregated states. Capturing the earliest structural transitions—prior to oligomer formation—remains challenging but is crucial for understanding the initial steps of the aggregation pathway. Time‐resolved approaches capable of probing αSyn's conformational landscape on sub‐second timescales are therefore essential to reveal the metal‐induced structural dynamics that precede aggregation.

Understanding how disease‐related factors, particularly metal ion interactions, influence the conformational ensemble of αSyn is crucial for elucidating its role in PD pathogenesis. In this study, we employ time‐resolved SAXS (TR‐SAXS) to capture the rapid, milliseconds structural changes of αSyn upon binding with transition metal ions. By capturing these early monomeric conformational shifts, such as elongation and compaction, we aim to identify structural intermediates that precede aggregation, thus shedding light on the molecular mechanisms driving metal‐induced αSyn aggregation. These insights not only enhance our understanding of αSyn's aggregation pathways but may also inform strategies for therapeutic intervention targeting early‐stage misfolding events.

## 2 Results

All IDPs are very difficult to target and investigate, due to their lack of a stable structure. Nevertheless, IDP structures may not be truly random because side‐chain steric interference and long‐range interaction may favor certain conformations. To gain insight into conformational dynamics of WT‐αSyn in the presence of transition metal ions (Mn^2^^+^, Fe^3^^+^, Cu^2^^+^, and Zn^2^^+^), we employed TR‐SAXS in a microfluidic mixing setup. We utilized a cross‐shaped microfluidic chip operating under laminar co‐flow, with the protein stream flanked by two buffer streams containing the metal ion of interest (Figure 1b). To assess the efficiency of mixing in our laminar flow system, we calculated the characteristic diffusion time for the small metal ions following Fick's law of diffusion using the diffusion coefficient 6.88 × 10^−10^m^2^/s (Mn^2+^), 6.05 × 10^−10^m^2^/s (Fe^3+^), 7.33 × 10^−10^m^2^/s (Cu^2+^), and 7.15 × 10^−10^m^2^/s (Zn^2+^). The calculated time for a metal ion to traverse the 50 µm from the channel wall to the center is approximately 1.75 s, corresponding to an axial distance of approx. 40 mm. This confirms that metal binding is initiated rapidly at the junction and is complete across the channel width within the total experimental observation time of 3.64 s, ensuring that the observed SAXS data report on the structural kinetics of a homogeneous reaction and are not convoluted with the ongoing mixing process. WT‐αSyn was introduced through the middle channel of the microfluidic chip and diluted via laminar flow focusing with HEPES buffer to a final concentration of 180 µm protein. While homogeneous metal distribution is achieved after the first few data points, WT‐αSyn diffuses more slowly. To confirm homogeneous distribution of the proteins, we tracked the forward scattering intensity I(0), which is proportional to protein concentration (Figure S1). The normalized signal, I(0)/I(0)_0_, decayed to a plateau of approx. 0.33 after ∼2.8 s, confirming the establishment of a homogeneously diluted protein solution (180 µm). Crucially, significant changes in the SAXS profiles were already observable within the first 0.5 s, long before the solution was fully homogenized. This demonstrates that the initial binding and conformational changes occur on a timescale faster than physical dilution. Moreover, the similar diffusion coefficients of the four metal ions suggest that the concentration gradients of both αSyn and the ions are comparable across the microfluidic device, providing a consistent background for comparing αSyn structural dynamics. The feasibility of the microfluidic setup was validated by using BSA (Bovine Serum Albumin) and SDS (Sodium Dodecyl Sulfate), which both diffuse much slower than αSyn and metal ions [47]. Our SAXS experiments provide a comparable view of the millisecond‐scale dynamics of αSyn in the presence of metal ions, but the microfluidic device could be further optimized in future studies to ensure more thorough mixing prior to SAXS measurements.

**FIGURE 2:**
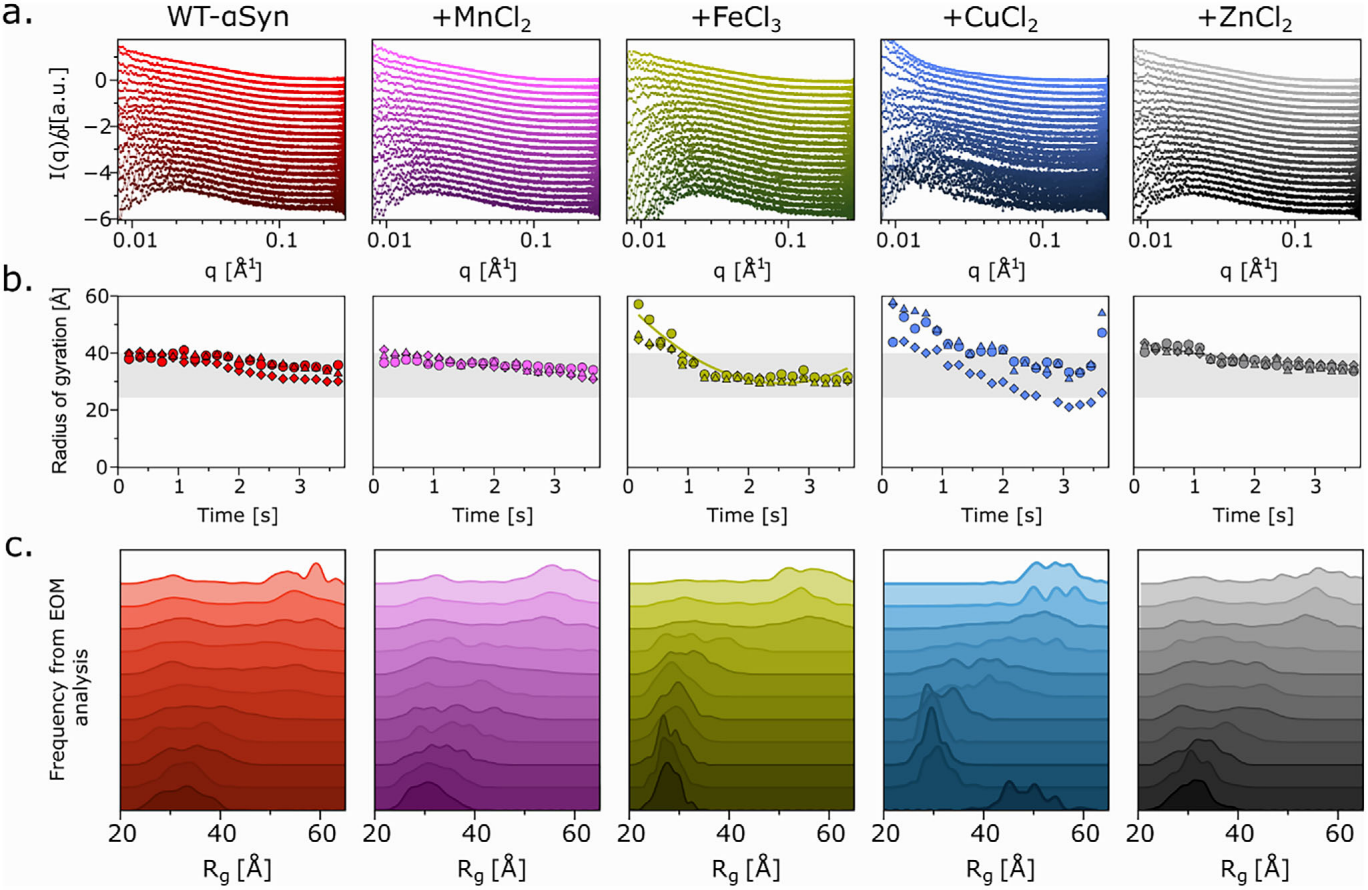
Changes in R_g_ after mixing with metal ions. (a) TR‐SAXS‐spectra (starting from top to bottom) after mixing αSyn with transition metal ions (molar ratio 2:1). (b) Averaged R_g_ values calculated using Guinier fitting (triangle Δ), SASonIDPs (lozenge ⬨), and GNOM (circle°) and (c) R_g_ distributions over time (starting from top to bottom) calculated by EOM. While only iron and copper influence the R_g_s, EOM indicates different dynamics after mixing.

To eliminate potential pre‐aggregates, the sample was prepared after size exclusion chromatography and centrifuged before loading. HEPES buffer was used, because it binds only weakly to metal ions with a very low precipitation risk. SAXS spectra were recorded at defined locations on the microfluidic chip, resulting in an experimental time of 3.64 s with a time resolution of 0.18 s, with buffer subtraction applied to eliminate any scattering contributions from the microfluidic channel. The SAXS data (Figure 2a) revealed differences in the scattering profiles of αSyn in the presence of different metal ions (ratio protein:metal ion, 2:1), reflecting changes in its conformational ensemble.

### 2.1 Radius of Gyration (R_g_) Analysis

To quantify changes in the overall size of WT‐αSyn, we calculated the radius of gyration (R_g_) for each metal condition using both Guinier fitting and GNOM analysis (Figure 2b). The Guinier fitting was performed through normal Guinier Fitting, fitting the low‐q region of the scattering curve with the Guinier equation (triangles) and by using the Sosnick Lab's SASonIDPs server [48, 49]. While the Guinier fitting is a simple and fast approach, it is limited to monodisperse, not flexible samples. The SAXSonIDPs server uses a modified Guinier model tailored for IDPs incorporating corrections based on polymer physics and provides a more accurate R_g_ for IDPs than normal Guinier fitting. Both methods use the reciprocal space, so might miss details of shape, therefore, we performed an indirect Fourier transform though GNOM analysis using the entire I(q) range to compute the real‐space P(r) distribution (pair‐distance distribution function). The results showed that, for WT‐αSyn, R_g_ only slightly decreased over time, indicating minor compaction after dilution. In contrast, metal ions altered the R_g_ to varying extents depending on the ion. Mn^2+^ and Zn^2+^ had a minimal impact on R_g_, while Cu^2+^ and Fe^3+^ led to an initial increase in R_g_ to values between 45–60 Å. Notably, Fe^3+^ induced a significant compaction of αSyn, reducing R_g_ to 31 Å, which was sustained throughout the experiment. Cu^2+^, however, leads to smaller R_g_ values when calculated using the Sosnick Lab's SASonIDPs server, suggesting a more compact structure or weak low‐q data. Unlike the other metal ions, Cu^2+^ ions appear to degrade low‐q data quality. This can either happen due to the strong absorption of X‐rays, different scattering, and enhanced radiation damage. Apart from Cu^2+^, the R_g_ values obtained by the two different methods are similar.

### 2.2 Ensemble Optimization Method (EOM) Analysis

Because the informative value of R_g_ is limited, we applied EOM to analyze the conformational distribution of αSyn over time for every second time point (Figure 2c). EOM is designed to model conformationally flexible proteins, by generating a large pool of random conformations based on the amino acid sequence and selecting an ensemble that best fits the experimental SAXS curve [2]. EOM revealed that αSyn adopts two predominant conformations initially: a compact form with R_g_ approx. 30 Å and an elongated form with R_g_ between 50–60 Å. Over time, the frequency of elongated conformations decreased, resulting in a more compact conformational distribution (25–40 Å). In the presence of Mn^2+^ or Zn^2+^, this trend was similar to WT‐αSyn. However, Cu^2+^ and Fe^3+^ exhibited distinct behaviors. Cu^2+^ causes a shift to more elongated conformations (47‐60 Å) at early timepoints, which compacted over time, but elongation persisted. This may reflect the conformational heterogeneity and a dynamic equilibrium between extended and compact states of monomeric αSyn, revealing a distinct structural fingerprint associated with copper–αSyn interactions. Fe^3+^, on the other hand, lead as well to an elongated conformation (49–63 Å) but then immediately shifted αSyn to compact states (R_g_ 23–36 Å), with no intermediate conformations observed. This suggests that Fe^3+^ promotes rapid compaction, while Cu^2+^ induces a more gradual transition to compact states characterized by a dynamic equilibrium. The elongated population of Cu^2+^ at the final time point coincides with increased noise and a degradation of low‐q data quality in the corresponding scattering curve. This increases uncertainty in the EOM‐derived population fractions, even though the χ^2^ value is acceptable (1.62).

The χ^2^ values of the EOM final ensembles were on average 1.19 ± 0.22 (WT‐αSyn), 1.45 ± 0.47 (Mn^2+^), 2.18 ± 0.79 (Fe^3+^), 3.33 ± 4.47 (Cu^2+^), and 1.49 ± 0.39 (Zn^2+^), respectively. Particularly the first two points impair the values for Cu^2+^ and Fe^3+^ and drops below 2 if they are excluded.

### 2.3 Kratky Plot and Fractal Dimension (D_m_) Analysis

**FIGURE 3:**
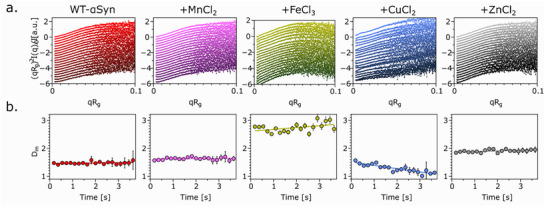
Iron and zinc lead to structural changes of αSyn. (a) Time resolved Kratky plots (from top to bottom) after mixing αSyn with transition metal ions (molar ratio 2:1). (b) Dm values obtained through fractal dimension fitting in the power‐law region over time.

The Kratky plot (Figure 3a) further revealed structural transitions in response to metal ion binding. WT‐αSyn displayed a typical IDP profile, with an extended tail indicative of flexibility. The presence of metal ions caused slight changes to this profile, which were quantified by fractal dimension (D_m_) fitting in the intermediate q‐region (power‐law) (Figure 3b). The fractal dimension reflects the compactness and internal organization of the protein, with lower values indicating more extended, flexible conformations and higher values corresponding to denser, compact structures. WT‐αSyn had an over time‐averaged D_m_ of 1.48 ± 0.04, consistent with an IDP in solution. Mn^2+^ led to a modest increase in D_m_ to 1.63 ± 0.05, suggesting slight compaction. Mn^2+^ is known to bind weakly [50], it might stabilize transient structured but does not drastically change folding. Zn^2+^ and Fe^3+^ caused more significant compaction with D_m_ values of 1.91 ± 0.05 and 2.74 ± 0.16, respectively. Unexpectedly, Cu^2+^ decreased the D_m_ to 1.28 ± 0.14, which appeared counterintuitive given its known aggregation‐promoting properties and the compaction observed through EOM. However, these two analyses probe different structural features. Whereas EOM reports global conformational distributions (R_g_), D_m_ reflects the internal mass density derived from the intermediate q‐region. Cu^2+^ may induces a heterogeneous ensemble in which elongated and partially compact states coexist, consistent with a dynamic equilibrium between extended and compact WT‐αSyn conformers. The low Dm therefore does not indicate global unfolding, instead, it suggests that the Cu^2+^‐bound ensemble remains internally loose or irregular, even when some compaction occurs at the R_g_ level. Thus, the EOM and D_m_ analyses are not contradictory but reveal complementary aspects of the structural heterogeneity associated with Cu^2+^‐WT‐αSyn interactions.

### 2.4 3D Structural Modeling and Clustering

**FIGURE 4:**
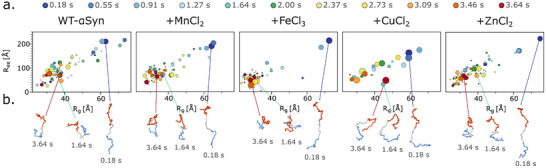
Conformations are getting denser over time. (a) Conformational landscapes projected onto R_g_ and R_ee_ of every second time point indicated by color from blue (0.18 s) to red (3.64 s). The fraction of the model is indicated by size. (b) Representative structures with the highest fraction of three timepoints are displayed below. The N‐terminal is colored in red, the NAC domain in grey, and C‐terminal in blue.

Using EOM, we generated low‐resolution, 3D models for each condition and analyzed their density and conformational landscape using R_g_ and end‐to‐end distance (R_ee_) (Figure 4). These models demonstrated that WT‐αSyn transitions from an elongated to a more compact structure over time. Mn^2+^ and Zn^2+^ had little effect on the conformational transition, while Fe^3+^ rapidly compacted αSyn, as seen in the narrowing of the R_g_ and R_ee_ distributions. Cu^2+^ led to more homogeneous distributions, suggesting a stable but heterogeneous population of conformers.

**FIGURE 5:**
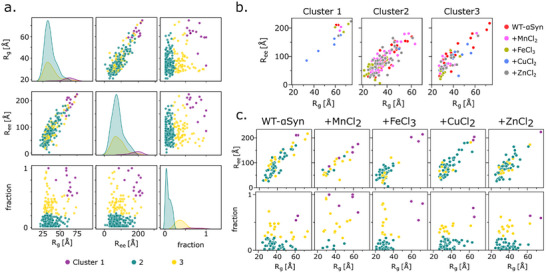
Transition metal ions modulate αSyn structural ensembles differently. (a) Hierarchical clustering of EOM‐derived conformations from 11 time points across all conditions revealed three major structural clusters: extended (cluster 1, purple), compact (cluster 2, teal), and intermediate (cluster 3, yellow). (b) Mean R_g_ and R_ee_ values of each cluster, with data points colored by metal ion condition. (c) Cluster distribution for WT‐αSyn and each metal treatment.

To further resolve the conformational heterogeneity and domain‐specific effects induced by metal ion binding, we performed hierarchical clustering on the final EOM ensembles across all conditions (Figure 5a). Three distinct structural clusters were identified: Cluster 1 featured extended conformations (average R_g_ = 60.9 Å, R_ee_ = 178.5 Å), cluster 2 comprised compact conformations (average R_g_ = 35.5 Å, R_ee_ = 79.1 Å), and cluster 3 captured intermediate states (R_g_ = 37.8 Å, R_ee_ = 86.6 Å). WT‐αSyn primarily occupied cluster 2 (68.9%), while Mn^2+^, Zn^2+^, and Fe^3+^ further stabilized compact states, increasing the proportion of cluster 2% to 75%, 71% and 73.2%, respectively. In contrast, Cu^2+^ induced a shift toward cluster 3, indicating more extended or partially folded intermediates that may predispose αSyn toward aggregation‐prone conformations.

### 2.5 Intra‐and Inter‐Domain Distance Analysis

**FIGURE 6:**
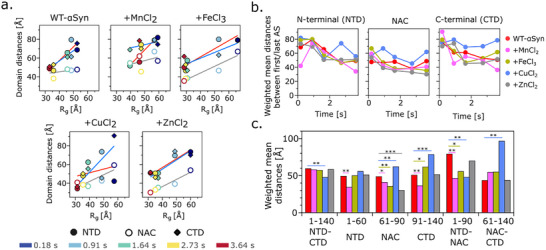
Transition metal ions alter intra‐ and inter‐domain distances in αSyn. (a) Weighted mean intra‐domain distances (NTD, (filled circle, ⬤) NAC (empty circle Ｏ), CTD (⬨) plotted against corresponding R_g_ values across, colored from early (blue) to late (red). (b) Time‐resolved evolution of average domain distances for each metal condition. (c) Domain distances at the final time point (3.64 s). Cu^2+^ uniquely expanded the CTD and inhibited domain compaction, while Zn^2+^ and Fe^3+^ promoted NAC and NTD rearrangements.

To assess how these conformational shifts affect domain flexibility, we calculated weighted mean intra‐domain distances for the NTD, NAC region and CTD at five time points post‐mixing (Figure 6a,b). The domain distances were calculated on the C^α^–C^α^ of the amino acids on the defined positions. While WT‐αSyn exhibited gradual compaction of the NTD and CTD over time, the NAC region remained relatively stable. Notably, Fe^3+^ induces rapid global compaction (see Figures 2 and 4), but paradoxically expanded the CTD slightly while compacting the NAC and promoting NTD‐NAC interaction. Mn^2+^ and Zn^2+^ both accelerated domain compaction, with Zn^2+^ showing pronounced effects on the NAC region. In contrast, Cu^2+^ disrupted this trend: it inhibited compaction of all three domains, significantly expanded the CTD (p<0.001), and reinforced NTD‐NAC proximity. These findings suggest that transition metal ions differentially modulate the conformational landscape of αSyn, with Cu^2+^ uniquely promoting extended or heterogeneous species that may represent aggregation‐prone states.

## 3 Discussion

Understanding the conformational dynamics of WT‐αSyn is crucial to unraveling the molecular mechanisms underlying its pathological aggregation in synucleinopathies such as Parkinson's disease. Here, we applied TR‐SAXS in a microfluidic setup to investigate how four different transition metal ions (Mn^2^^+^, Fe^3^^+^, Cu^2^^+^, and Zn^2^^+^) influence the structural ensemble of monomeric αSyn on a sub‐second timescale. Our results reveal distinct and metal‐specific effects on the compaction, domain interactions, and conformational heterogeneity of WT‐αSyn, offering new insights into early‐stage conformational shifts that may be relevant to pathogenic aggregation.

Among the metals tested, Fe^3^^+^ had the most pronounced impact. It induced an immediate and sustained compaction of the WT‐αSyn ensemble, reducing the radius of gyration (R_g_) to as low as 31 Å. EOM analysis showed a strong reduction in the population of elongated conformation within milliseconds of mixing, with conformational landscapes dominated by compact models and a shift toward cluster 2 (compact structures). The observed increase in fractal dimension (D_m_) to 2.74 further supports the notion that Fe^3^^+^ stabilizes denser, more space‐filling conformations. Intriguingly, although Fe^3^^+^ promoted globular compaction, intra‐domain analysis revealed a paradoxical elongation of the CTD, alongside compaction of the NAC domain and tighter N‐terminal‐NAC interactions. These findings suggest that Fe^3^^+^ binding may induce a reorganization of intramolecular contact, potentially consistent with structural rearrangements that could precede nucleation or early oligomer formation [29, 51]. Interestingly, when we increased the metal ion concentration to a molar ratio of 1:1, Fe^3^^+^ ion leads to the formation of oligomers (R_g_ = 150 Å) immediately after 0.2 s (see Figure S1). Interestingly, even though Fe^3^^+^ ion seems to alter the early aggregation kinetics, a cryo‐EM study revealed that the fibril polymorph is not changed [37].

Cu^2^^+^ exhibited an opposing and more complex behavior. It initially promoted elongated conformations and reduced D_m_, suggestive of an expanded, less compact ensemble. Over time, EOM analysis showed a modest shift toward more compact conformers, as observed due to computational analysis [52], but Cu^2^^+^‐treated WT‐αSyn retained broader and more heterogeneous R_g_ distributions than other conditions. Clustering results indicated a dominant population in cluster 3 (intermediate conformations), while intra‐domain measurements revealed significant expansion of the CTD and no compaction of the NTD or NAC regions. These features are consistent with Cu^2^^+^ being associated with extended or partially structured states that may expose aggregation‐prone regions. Notably, Cu^2^^+^ is known to bind the WT‐αSyn N‐terminal region with high affinity and has been shown to accelerate amyloid formation [53], possibly though stabilization of partially folded intermediates or promotion of long‐range contacts between flexible parts. In our TR‐SAXS experiments, Cu^2+^ induces early elongation followed by partial compaction, with the final time point showing in apparent increase in elongated conformers, likely due to reduced data quality. Importantly, these experiments were performed at a 2:1 protein:metal ratio to maintain monomeric conditions and avoid rapid oligomerization. Under these conditions, partial occupancy and heterogeneous binding likely contribute to the observed structural diversity.

While these results reveal clear Cu^2+^‐dependent structural heterogeneity, the specific molecular determinants underlying these conformational shifts remain unresolved. Cu^2+^ residues have been reported to interact with residues across the NTD and CTD, suggesting that multiple low‐ and high‐affinity sites contribute cooperatively to the observed ensemble changes. However, the current data do not allow assignment of structural effects to individual residues or domains. Future mechanistic studies involving site‐directed mutagenesis will be essential to determine which binding site drives elongation, partial completion, or heterogeneity.

Mn^2^^+^ and Zn^2^^+^ both induced milder and more progressive compaction, generally mirroring the behavior of WT‐αSyn over time. Mn^2^^+^ led to subtle increases in D_m_ and reinforced NTD and NAC interactions without major changes in overall R_g_ distributions. Zn^2^^+^, in contrast, more significantly affected the NAC region and contributed to domain‐specific compaction, while also increasing occupancy in compact cluster 2. These findings align with the literature, in which Mn^2^^+^ is considered a weak binder with minimal impact on aggregation [28, 50, 54], whereas Zn^2^^+^ has been reported to induce modest structural changes in WT‐αSyn and influence membrane binding or fibrillation under certain conditions [36, 55]. We also observed compaction of the CTD consistent with reported C‐terminal binding of Mn^2^^+^ [28, 56].

All time‐resolved SAXS experiments were performed at a 2:1 protein:metal ion ratio, which was selected to preserve monomeric scattering profiles and avoid rapid aggregation observed at equimolar or higher metal concentrations. Preliminary static SAXS trials revealed that equimolar (metal ion: protein, 1:1) conditions for Fe^3^^+^ (Figure S2) and 1:10 for Mn^2^^+^ (Figure S3) caused immediate oligomer formation, preventing reliable time‐resolved measurements. Furthermore, static SAXS measurements collected ∼10 min after sample loading (Figure S4) demonstrated that all tested metal ions promote αSyn aggregation at a 1:1 molar ratio or higher relative to the protein. While the 2:1 ratio condition enabled consistent time‐resolved measurements across different ions, it represents a key limitation of the present study. Under sub‐stoichiometric condition, the ensemble necessarily includes a mixture of metal‐bound and unbound αSyn molecules, and the resulting conformational heterogeneity cannot be uniquely attributed to a single binding state. Aggregation observed at higher metal ratios therefore contains the accessible experimental regime but does not explain the behavior observed at 2:1. Consequently, the ion‐specific conformational dynamics reported here should be interpreted as ensemble‐level response within a stable, partially occupied regime. Systematic investigation of stoichiometry‐dependent effects will require complementary approaches capable of probing higher metal loadings without inducing aggregation. In addition, several of the investigated ions, most notably Cu^2^^+^ and Fe^3^^+^, are known to exhibit heterogenous binding affinities, multi‐site coordination and long‐range electrostatic interactions with αSyn, while even a single ion can remodel the structural ensemble without requiring full 1:1 occupancy [28, 37], this binding complexity further limits the extent to which a complete mechanistic picture can be derived from a single fixed 2:1 ratio alone.

Together, these results illustrate that the structural ensemble of monomeric WT‐αSyn is sensitive to metal ion binding, with each ion inducing a distinct kinetic and structural fingerprint. The effects of metal ions on WT‐αSyn conformations reflect their chemical properties and electronic configuration. Fe^3^^+^, with high charge density and octahedral coordination, rapidly stabilizes compact states, which may correlate with early toxic oligomer formation, whereas Cu^2^^+^, with a flexible d^9^ coordination, promotes heterogeneous, partially folded species, possibly representing aggregation‐prone intermediates. Mn^2^^+^ and Zn^2^^+^, with less specific or weaker binding, support more modest compaction trajectories, suggesting limited roles in initiating aggregation directly.

This work demonstrates the utility of TR‐SAXS combined with ensemble modeling to capture conformational transitions in disordered proteins under near‐physiological conditions. Importantly, the real‐time resolution of conformation changes allows us to dissect early structural shifts that may precede irreversible aggregation events. Nevertheless, the interpretation of certain features (especially for Cu^2^^+^) must be made cautiously due to potential complications from radiation damage, low‐q scattering degradation, and ion‐induced changes in sample stability. Further studies combining SAXS with orthogonal methods such as NMR, FRET, or single‐molecule spectroscopy would help refine mechanistic insights. Furthermore, how the interaction is leading to ROS production and metal ion reduction should be pursued further.

In conclusion, our findings support the view that transition metal ions modulate the conformation ensemble of WT‐αSyn in distinct and functionally relevant ways. These early structural perturbations may seed or bias aggregation pathways, offering mechanistic links between metal dyshomeostasis and synucleinopathies. Targeting metal‐induced conformation transitions could represent a potential therapeutic avenue to prevent or delay αSyn misfolding and toxicity.

## 4 Conclusion

In this study, we used time‐resolved SAXS to capture the earliest conformational responses of monomeric WT‐αSyn to physiologically relevant transition metal ions. Our results reveal that each ion induces a distinct structural fingerprint. Fe^3^^+^ drives rapid and pronounced compaction, Cu^2+^ promotes a heterogeneous ensemble with early elongation and partial late‐stage compaction, while Mn^2^^+^ and Zn^2^^+^ cause only modest structural rearrangements. These findings highlight how metal‐specific coordination chemistry, binding affinity, and charge density shape the conformational landscape of WT‐αSyn on sub‐second timescales. By resolving these early structural transitions, our work provides mechanistic insight into how metal interrupted homeostasis may bias aggregation pathways linked to Parkinson's disease. Together, these results underscore the value of TR‐SAXS for dissecting rapid structural events in intrinsically disordered protein and lay the groundwork for future integrative studies of meal‐induced misfolding.

## 5 Experimental Section/Methods

### 5.1 Protein Sample Preparation

WT‐αSyn was expressed in BL21(D3) E. Coli cells and purified as described previously. Briefly, the expression was conducted overnight at 20∼C in LB‐medium and harvested. The protein was precipitated using 1.75 m ammonium sulphate after lysis of the cell pellet by heat and ultrasonication (17% amplitude, 5 min, 5 s off/3 s on). Nucleic acid impurities were removed through streptomycin sulphate precipitation. The protein was re‐suspended in 25 mm Tris‐HCl, pH 8, filtered (0.42 pore size) and subsequently loaded onto a HiTrapQHP anion exchange chromatography column (GE Healthcare). The protein was eluted using a gradient with 25 mm Tris‐HCl, pH 8, 800 mm NaCl, and the αSyn containing fractions were combined, precipitated with ammonium sulphate, and the pellet was stored at −20°C. The pellet was re‐suspended in 25 mm Tris‐HCl, pH 7.5 containing 6 m Guanidium chloride, and size exclusion chromatography was performed using a HiLoad 16/600 Superdex 75 pg at a flow rate of 0.750 mL/min. αSyn concentration was determined by measuring UV‐absorption at 280 nm (extinction coefficient of 5960 m‐1cm‐1), aliquoted and lyophilised after dialysis against ammonium bicarbonate.

### 5.2 Time‐Resolved Small Angle X‐ray Scattering (TR‐SAXS) Measurement

To understand the impact of transition metal ions on the monomeric structural dynamics, we performed time‐resolved small‐angle X‐ray scattering experiments using an adaptive microfluidic cross‐shaped channel chip (Microfluidic ChipShop, Germany, 10000258, topas material) with the following dimensions: channel depth: 100 µm, channel width: 100 µm, and channel length: 87 mm. All three channels are syringe pump driven with a total flow rate of 14 µl/min. The middle channel was used for the protein solution and both side channels were used for buffer alone or buffer containing transition metal ions. To estimate the diffusion‐limited mixing of metal ions in the microfluidic channel, we used diffusion coefficients for aqueous metal cations at infinite dilution taken from the aqion database: 6.88 × 10^−10^m^2^/s (Mn^2+^), 6.05 × 10^−10^m^2^/s (Fe^3+^), 7.33 × 10^−10^m^2^/s (Cu^2+^) and 7.15 × 10^−10^m^2^/s (Zn^2+^). We then applied Fick's law of diffusion by computing the characteristic diffusion time t_D_ = y^2^/(2D), where y is the half‐width of the channel (50 µm), to estimate the time and downstream distance required for these ions to diffuse across the channel width. The protein uniform distribution was determined by monitoring the normalized scattering intensity I(0)/I(0)_0_.

Since an increase in particle radius by a factor of 10 would increase the signal intensity at zero angle by a factor for 10^6^, potential pre‐aggregates were removed by centrifugation prior to the measurement.

The SAXS measurement was performed at the coSAXS beamline at MAX IV, Lund, Sweden, equipped with an Eiger2 4 m SAXS detector. SAXS data (I(s) vs. q, where q = 4πsinθ/λ, 2θ is the scattering angle, and λ = 0.124 nm is the wavelength) were recorded at room temperature as 20 × 20 ms frames, 50 repetitions for each time point. The energy was 12.4 keV and the focusing distance 3.5 m. The beam size was 128 µm width and 80 µm height.

Subtraction and data processing were performed using the ATSAS software package including PRIMUS [57].

The protein and transition metals were mixed to a final molar ratio of 2:1 (180 µm:90 µm).

Radius if gyration (R_g_) were calculated by performing normal Guinier fitting using PRIMUS, a modified Guinier model tailored for IDPs incorporating corrections based on polymer physics using the Sosnick Lab's SASonIDPs server [48, 49], and the pair‐distance distribution function using GNOM [58].

Fractal dimension fitting was performed by fitting the negative slope of the linear region in a log(I) vs. log(q), using the following equation: log(I) = log(I_0_)‐D_m_ log(q). The linear region of the power‐law region (intermediate‐q), where the intensity follows I(q)∼q^−Dm^, was used for the fractal fitting (around log(q)≈‐1.4 to ‐1.

### 5.3 EOM Analysis

Ensemble Optimization Method (EOM) 3.2.1 [2, 59] was applied to fit the experimental SAXS data to an averages theoretical scattering intensity derived from an ensemble of conformations based upon the WT a‐Syn sequence alone. Completely disordered configurations of the alpha‐carbon trace were used without the input of rigid bodies. Initially, 100 000 models were generated with RANCH and the theoretical scattering intensities of the models in the pool were computed using FFMAKER. A genetic algorithm (GAJOE) was applied for the selection of an ensemble. 1000 generations with 50 ensembles were run. The final ensemble size was fixed to 50 curves per ensemble and the background was adjusted to a constant.

The R_g_ distributions and final ensemble were investigated into more detail.

The models of the final ensembles were visualized using UC SF ChimeraX version 1.9 [60, 61, 62] coloring the N‐terminal domain (NTD), NAC region, and C‐terminal domain (CTD) separately. The Intra‐and inter‐domain distances were measured using PyMOL, version 3.0, Schrödinger. The distances of the C^α^–C^α^ of the following amino acids were measured: 1 and 140 (end‐to‐end distance (R_ee_), distance between NTD and CTD), 1 and 60 (distance within NTD), 61 and 90 (distance within CTD), 91 and 140 (distance within CTD), 1 and 90 (distance between NTD and NAC), and 61 and 140 (distance between NAC and CTD). The weighted mean according to the ratio within the final ensemble was calculated for each distance at the specific time point.

### 5.4 Hierarchical Clustering

Structural ensembles were analyzed across five conditions (WT‐αSyn, and in the presence of Mn^2^^+^, Cu^2^^+^, Fe^3^^+^, and Zn^2^^+^) using a hierarchical clustering approach implemented in Python. For each system, we used the radius of gyration (R_g_), end‐to‐end distance (R_ee_), and ratio from the final ensembles of the EOM analysis. The features were standardized and clustered using Ward's linkage with Euclidean distance, with parallel analyses performed on both raw and weighted features. The optimal cluster count (k+3) was determined through dendrogram inspection and validated via silhouette analysis. Cluster assignments were generated using a distance threshold of t = 2.5, with statistical significance of condition‐dependent differences assessed via Kruskal‐Wallis and post‐hoc Dunn's tests (α = 0.05, Benjamini‐Hochberg corrected). Effect sizes were quantified using Hedge's g, with |g|>0.8 considered biologically significant. Visualizations were created using Matplotlib, Seaborn, and DataGraph, showing pairwise relationships between R_g_, R_ee_ and fraction within conditions and across conditions within clusters.

## Funding

This work has been supported by the Swiss National Scientific Foundation (project ID: 310030_197626 and 10002967, J.L.).

## Conflicts of Interest

The authors declare no conflicts of interest.

## Supporting information




**Supporting File**: advs74508‐sup‐0001‐SuppMat.docx.

## Data Availability

The data that support the findings of this study are available from the corresponding author upon reasonable request.
