# Supplementary material for: Time‐Resolved SAXS Reveals Distinct Millisecond Metal‐Induced Conformational Dynamics of Monomeric α‐Synuclein
Source: Adv Sci (Weinh). 2026 Feb 27;13(25):e12293. doi: 10.1002/advs.202512293 (PMC13137826; doi:10.1002/advs.202512293)
Supplement: Supplementary file 1 — Supporting File: advs74508‐sup‐0001‐SuppMat.docx. [file ADVS-13-e12293-s001.docx]

Supporting Information

Time-Resolved SAXS Reveals Distinct Millisecond Metal-Induced Conformational Dynamics of Monomeric α-Synuclein

*Rebecca Sternke-Hoffmann^1^, Miriam Dos Santos Pinto^1^, Xue Wang^1^, Jinghui Luo^1*^*

^1^PSI Center for Life Sciences, 5232 Villigen PSI, Switzerland

*Corresponding author: Jinghui Luo ([Jinghui.luo@psi.ch](mailto:Jinghui.luo@psi.ch)).


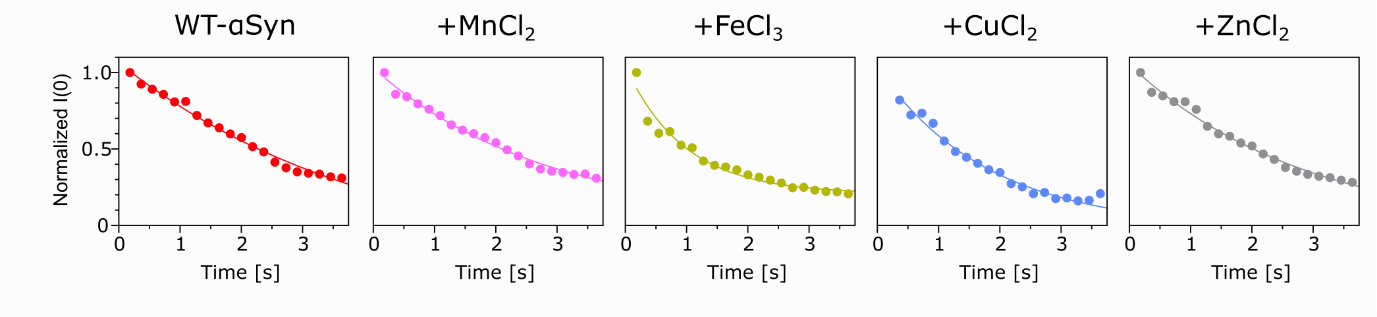


**Figure S1: Normalized forward scattering intensity used to judge the homogenous mixing.** Normalized forward scattering intensity I(0)/I(0)_0_, where I(0)_0_ is the intensity at the initial measurement point, is plotted against experimental time. The decay reflects both protein dilution and metal-induced structural transitions. Homogeneous mixing is achieved after approx. 2.8 s (I(0)/I(0)_0_≈0.33). Apparent rate constant obtained from exponential fitting reveal distinct metal-specific behaviors. WT-αSyn (0.24 s^-1^) shows baseline dilution kinetics, while Mn^2+^ (0.36 s^-1^) and Zn^2+^ (0.34 s^-1^) cause modest acceleration. In contrast, Cu^2+^ (0.55 s^-1^) and particularly Fe^3+^ (1.03 s^-1^) induce substantially faster kinetics, indicating rapid metal-induced compaction and oligomerization.


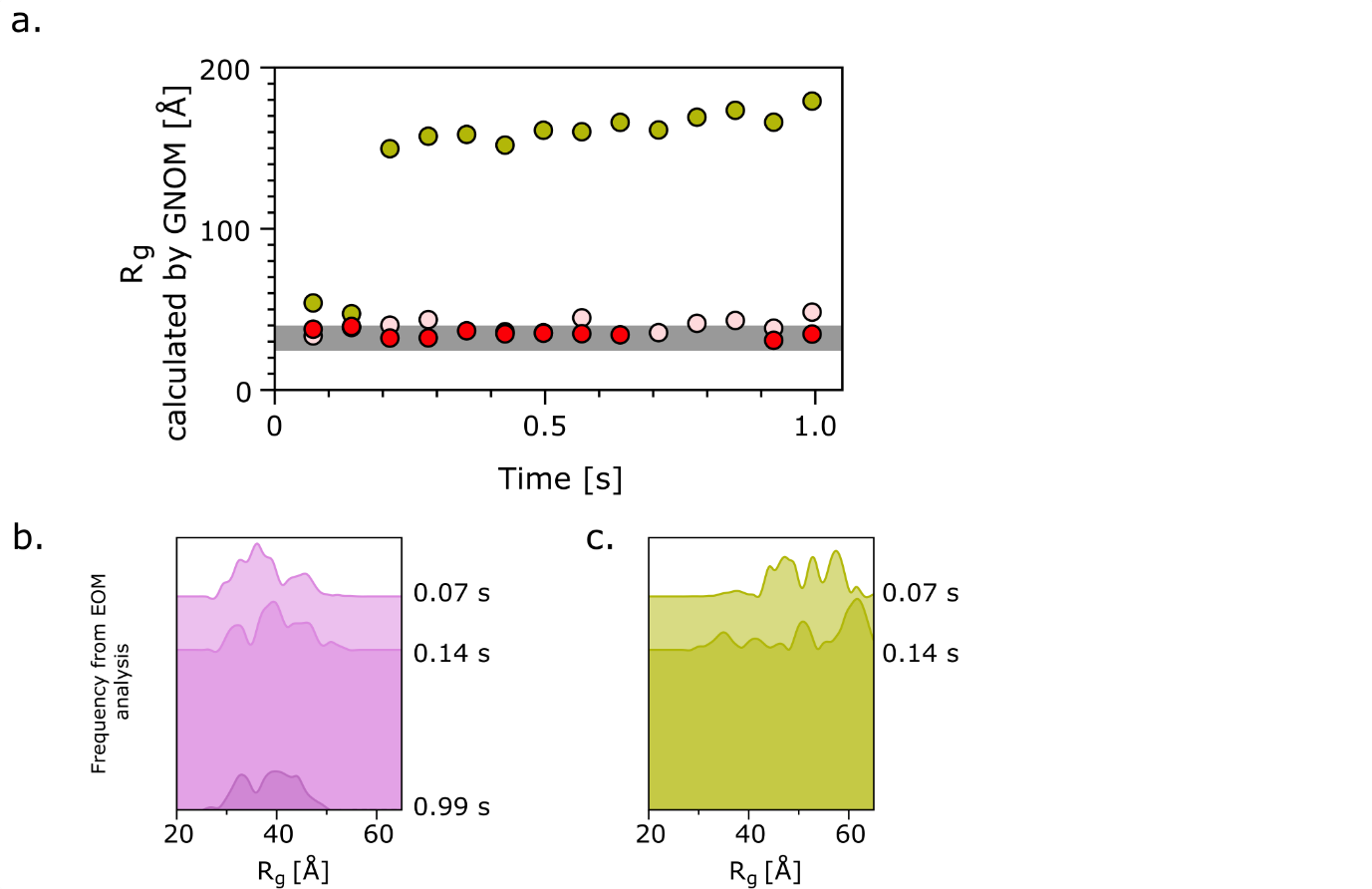


**Figure S2: Higher concentration of iron lead to a fast formation of aggregates**. **(a)** αSyn was mixed with FeCl_3_ and MnCl_2_ to a final molar ratio of 1**:**1. While 1**:**1 Mn^2+^ is not influencing the R_g_, similar to behavior observed for 2**:**1 (protein**:**metal ion), induces the higher Fe^3+^ concentration immediate aggregation. R_g_ distributions over time calculated by EOM in the presence of **(b)** Mn^2+^ and **(c)** Fe^3+^. EOM could only be applied for the first two data points for Fe^3+^.


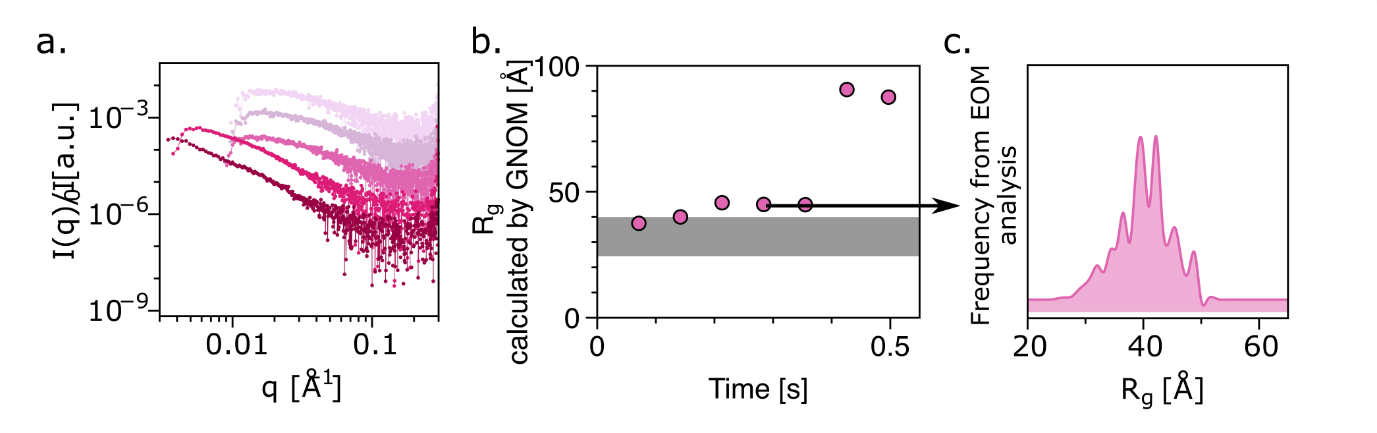


**Figure S3: High MnCl_2_ concentration led to formation of aggregates**. **(a)** SAXS-spectra of αSyn mixed with MnCl_2_ to a final molar ratio of 1**:**10 (protein:metal ion) (from top to bottom) and **(b)** the corresponding R_g_ calculated by GNOM. **(c)** R_g_ distribution after 0.3 s calculated by EOM with a χ^2^ of 5.4.


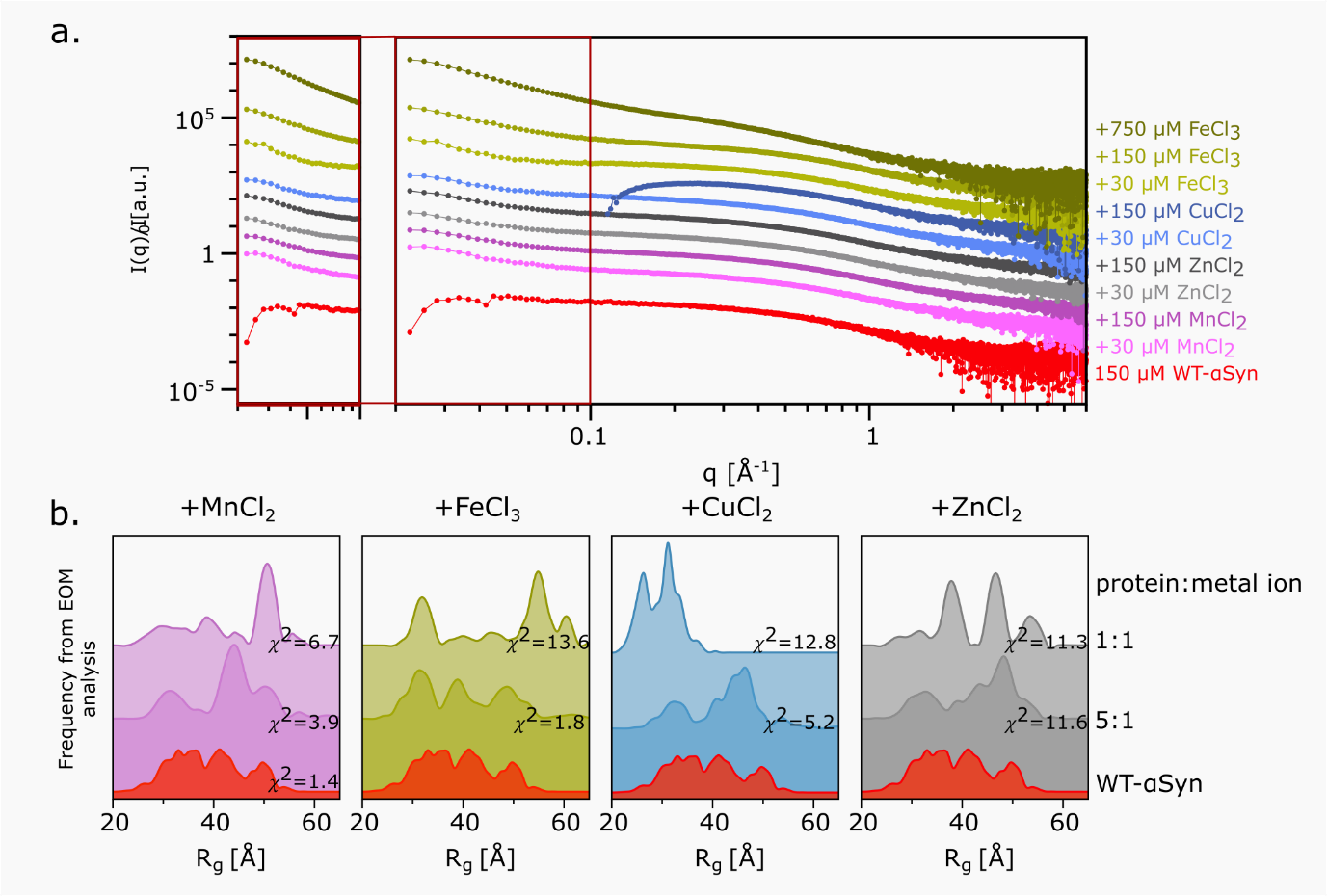


**Figure S4: Static SAXS measurement in the presence of different metal ion concentrations. (a)** 150 µM WT-αSyn was measured after mixing with different metal ions. The sample was prepared onto PCR stripes and loaded into the beam using an automated sample changer leading to several minutes of incubation before measurement. The low-q area is highlighted on the left to emphasize on the aggregation **(b)** R_g_ distributions calculated by EOM of WT-αSyn with the metal ion in a molar ratio of 1:1 and 5:1 (protein:metal ion). The corresponding χ^2^ values are displayed, proving aggregation and the impossibility to analyze most of the samples using EOM.
